# Diagnostic Stratification of Pancreatic Ductal Adenocarcinoma via Metallomics and Blood-Based Biomarkers

**DOI:** 10.3390/diagnostics15212818

**Published:** 2025-11-06

**Authors:** Donatella Coradduzza, Teresa Perra, Leonardo Sibono, Andrea Sanna, Maurizio Cossu, Emanuela G. Azara, Francesco Petracca, Roberto Beniamino Madeddu, Maria Rosaria De Miglio, Ciriaco Carru, Massimiliano Grosso, Maria Laura Cossu, Serenella Medici

**Affiliations:** 1 Department of Biomedical Sciences, University of Sassari, 07100 Sassari, Italy; rmadeddu@uniss.it (R.B.M.); carru@uniss.it (C.C.); 2 Department of Medical, Surgical and Experimental Sciences, University of Sassari, Viale San Pietro 8, 07100 Sassari, Italy; tperra@uniss.it (T.P.); francescopetracca09@gmail.com (F.P.); demiglio@uniss.it (M.R.D.M.); mlcossu@uniss.it (M.L.C.); 3 Department of Mechanical, Chemical, and Materials Engineering, University of Cagliari, 09124 Cagliari, Italy; leonardo.sibono@unica.it (L.S.); massimiliano.grosso@unica.it (M.G.); 4 SC Chimica Istituto Zooprofilattico Sperimentale della Sardegna, Via Duca degli Abruzzi, 8, 07100 Sassari, Italy; andrea.sanna@izs-sardegna.it (A.S.); maurizio.cossu@izs-sardegna.it (M.C.); 5 Institute of Biomolecular Chemistry, National Research Council, 07100 Sassari, Italy; e.azara@icb.cnr.it; 6 Unit of Oncology, University Hospital of Sassari, 07100 Sassari, Italy; 7 Department of Chemistry and Pharmacy, University of Sassari, 07100 Sassari, Italy; sere@uniss.it

**Keywords:** diagnostic biomarkers, pancreatic ductal adenocarcinoma, inflammation indices, clinical biochemistry, patient stratification ICP-MS, metallomics

## Abstract

**Background:** Pancreatic ductal adenocarcinoma (PDAC) remains one of the deadliest cancers, largely due to late diagnosis and the lack of reliable non-invasive biomarkers. Altered trace element homeostasis has been implicated in tumor biology and systemic inflammation, but comprehensive metallomic profiling in PDAC is still limited. **Methods:** Using inductively coupled plasma mass spectrometry (ICP-MS), we quantified 20 serum and 15 urinary metals in 71 PDAC patients and 69 matched controls. Statistical analyses included univariate Wilcoxon testing, correlation with systemic inflammatory indices (NLR, MLR, SIRI, AISI, HGB/RDW, PCT), and multivariate chemometric modeling (PCA-LDA). K-means clustering was applied to identify patient subgroups with distinct biochemical signatures. **Results:** PDAC patients showed significantly elevated urinary antimony, chromium, cadmium, and vanadium, whereas controls exhibited higher serum selenium, zinc, barium, vanadium, and cobalt (all *p* < 10^−5^). The PCA-LDA model achieved 99% classification accuracy (Monte Carlo cross-validation, 1000 iterations), highlighting complementary diagnostic contributions of serum and urinary profiles. Serum selenium was inversely associated with SIRI and NLR, while urinary cobalt correlated positively with NLR. Clustering revealed three PDAC subgroups with different inflammatory and metallomic patterns, suggesting underlying biological heterogeneity. **Conclusions:** PDAC is characterized by opposite serum-urine metal signatures, indicating altered absorption-excretion dynamics. Selenium depletion may represent a protective biomarker, whereas urinary cobalt excretion reflects systemic inflammation. This integrative ICP-MS–chemometric approach provides a promising diagnostic tool for improving early detection and patient stratification in clinical practice.

## 1. Introduction

Pancreatic cancer (PC) is one of the most lethal malignancies worldwide, with a five-year survival rate of approximately 13%, according to the latest SEER and American Cancer Society reports (2025) [1,2]. This dismal prognosis is primarily attributable to late-stage diagnosis, the aggressive biological behavior of the tumor, and the limited efficacy of currently available treatments [3]. In 2024 alone, more than 66,000 new cases and over 51,000 PC-related deaths were projected in the United States, highlighting the urgent need for improved diagnostic strategies capable of enabling earlier detection and better patient outcomes [4].

Recent therapeutic advances have aimed to improve the dismal prognosis of pancreatic ductal adenocarcinoma (PDAC) through multimodal and molecularly targeted approaches. Conventional chemotherapy regimens such as FOLFIRINOX or gemcitabine/nab-paclitaxel remain the backbone of treatment for advanced disease, yet intrinsic and acquired chemoresistance continue to limit survival benefits. As reviewed by Peshin et al. [5], multidisciplinary management—including neoadjuvant strategies, adjuvant combination regimens, and precision chemotherapy—has reshaped therapeutic paradigms across resectable and metastatic stages. Emerging immunotherapeutic modalities, such as checkpoint inhibitors and mRNA-based vaccines, are also under active investigation.

Moreover, experimental targeted therapies have focused on molecular pathways that sustain PDAC progression and drug resistance. Focal adhesion kinase (FAK), a non-receptor tyrosine kinase implicated in adhesion, invasion, and survival, represents a promising therapeutic target; diamine-substituted 2,4-pyrimidine-based FAK inhibitors have shown potent antitumor effects and the ability to overcome chemoresistance [6]. Similarly, inhibition of TANK-binding kinase 1 (TBK1)—a downstream effector of the KRAS/NF-κB axis—has demonstrated significant anti-proliferative and pro-apoptotic activity in PDAC cell lines [7]. Collectively, these findings highlight a transition toward integrative, mechanism-based therapeutic strategies combining cytotoxic, immune, and targeted approaches to counteract PDAC’s intrinsic resistance and aggressive biology.

Among histological subtypes, pancreatic ductal adenocarcinoma (PDAC) accounts for more than 90% of cases. PDAC is characterized by rapid progression, profound metabolic reprogramming, and pronounced resistance to both chemotherapy and radiotherapy [8,9]. Current diagnostic tools, including imaging modalities and the serum biomarker carbohydrate antigen 19-9 (CA19-9), are hindered by insufficient sensitivity and specificity, particularly in the early stages of disease, and no reliable biomarkers have yet been validated for routine clinical use [10,11,12,13].

Emerging evidence suggests that both environmental exposures and systemic metabolic disturbances contribute to pancreatic carcinogenesis, with increasing attention directed toward the role of essential and toxic metals [14,15,16]. Essential elements such as selenium (Se), zinc (Zn), copper (Cu), iron (Fe), and manganese (Mn) are indispensable for critical physiological functions, including antioxidant defense, enzymatic catalysis, immune modulation, and DNA repair [17,18]. Maintaining their homeostasis is crucial for genomic stability and redox balance. Perturbations in these systems can impair metalloenzyme activity, reduce the efficiency of DNA repair mechanisms, and lead to the accumulation of oxidative DNA damage—events that facilitate malignant transformation. In contrast, toxic metals such as cadmium (Cd), lead (Pb), nickel (Ni), and chromium (Cr) are recognized for their carcinogenic potential, mediated through multiple interrelated biochemical pathways [19,20,21]. These include the generation of excessive reactive oxygen species (ROS) that damage DNA, lipids, and proteins; inhibition of DNA repair enzymes via direct binding to catalytic domains or displacement of essential cofactors; disruption of metalloprotein structure and function; and induction of epigenetic alterations, such as aberrant DNA methylation and histone modifications. For instance, Cd can mimic Zn within zinc-finger proteins, impairing their structural integrity and regulatory functions; Ni may induce promoter hypermethylation of tumor suppressor genes, silencing critical cell-cycle regulators; Cr(VI) generates highly reactive intermediates that promote DNA strand breaks and crosslinks; and Pb interferes with heme- and zinc-dependent enzymes, amplifying oxidative stress and genomic instability [22,23].

Metal dyshomeostasis may also contribute to the remodeling of the tumor microenvironment via chronic inflammation. Prolonged exposure to certain metals activates redox-sensitive transcription factors such as NF-κB and AP-1, driving the release of pro-inflammatory cytokines, prostaglandins, and nitric oxide [24]. These mediators collectively promote cellular proliferation, angiogenesis, and immune evasion, thereby sustaining a pro-tumorigenic environment. In PDAC, systemic inflammation is a well-recognized driver of disease progression, and hematological indices—including the neutrophil-to-lymphocyte ratio (NLR), monocyte-to-lymphocyte ratio (MLR), and systemic inflammation response index (SIRI)—have emerged as independent prognostic biomarkers. Nevertheless, the biochemical relationship between trace element dysregulation and systemic inflammatory status in PDAC remains largely unexplored [25,26].

In this context, metallomic profiling represents a promising approach to simultaneously quantify essential and toxic elements in biological fluids, offering a potential biochemical signature of disease state. Advances in inductively coupled plasma mass spectrometry (ICP-MS) now permit highly sensitive multi-elemental analysis in serum and urine, enabling the detection of subtle but biologically meaningful deviations in trace element concentrations [27]. Importantly, when integrated with systemic inflammatory indices and analyzed through advanced multivariate chemometric modeling, metallomic data can uncover complex multidimensional associations that remain undetectable with traditional univariate methods [28].

Recent investigations have expanded our understanding of metal dyshomeostasis in pancreatic ductal adenocarcinoma (PDAC) by linking elemental alterations to both diagnosis and prognosis. Byeon et al. conducted the first comprehensive analysis of the serum and tissue metallome in PDAC, revealing markedly decreased serum concentrations of magnesium, iron, zinc, and selenium, paralleled by opposite trends in tumor tissue. Notably, lower serum selenium and iron levels were significantly associated with poorer overall survival, suggesting that systemic-tumoral metal redistribution may serve as an early marker of disease aggressiveness [29].

In parallel, a growing body of evidence highlights the role of zinc homeostasis and its transporters in pancreatic carcinogenesis. ZIP4 (*SLC39A4*), the predominant zinc importer, is consistently overexpressed in PDAC and contributes to tumor growth, invasion, and chemoresistance by activating the CREB–STAT3–Cyclin D1 axis and the miR-373–PHLPP2–AKT–TGFβ signaling pathway [30]. Mechanistically, ZIP4 also regulates epithelial–mesenchymal transition (EMT) and metastatic plasticity via co-activation of the zinc-dependent transcription factors ZEB1 and YAP1, forming the ZIP4–miR-373–LATS2–YAP1–ITGA3 regulatory cascade [31]. Beyond pancreatic cancer, dysregulated zinc metabolism involving the SOX4-ZIP14 axis has been shown to promote oncogenesis and immune evasion through the creation of a zinc-deficient tumor microenvironment that suppresses CD8^+^ T-cell activity [32].

Collectively, these findings provide a strong biological framework supporting our approach to assess both essential and toxic metals in serum and urine, reinforcing the concept that zinc and selenium imbalance, coupled with inflammatory activation, represents a central hallmark of PDAC pathophysiology and a promising target for biomarker discovery.

The present study employed ICP-MS to perform a comprehensive analysis of 20 serum and 15 urinary metals in patients with histologically confirmed PDAC. Our objectives were to identify deviations from population reference values, to investigate correlations between elemental concentrations and systemic inflammatory indices (NLR, MLR, SIRI), and to apply multivariate and clustering analyses for patient stratification. By combining metallomics, clinical biochemistry, and statistical modeling, this integrative study aims to deepen understanding of the interplay between trace element imbalance and inflammation in PDAC, while contributing to the identification of novel diagnostic and prognostic biomarkers with potential clinical applicability.

## 2. Materials and Methods

### 2.1. Study Population and Patient Selection

The study population included patients with a histologically confirmed diagnosis of pancreatic ductal adenocarcinoma (PDAC), consecutively recruited at the University Hospital of Sassari (A.O.U. SS, Department of General Surgery) between September 2023 and March 2025. The study was designed to characterize the elemental profile and systemic inflammatory status of newly diagnosed PDAC patients before initiation of any anticancer therapy. Eligible participants were adults (≥18 years) whose diagnosis had been established through imaging modalities—computed tomography (CT), magnetic resonance imaging (MRI), or endoscopic ultrasound—and subsequently confirmed by histopathological examination.

Inclusion criteria required availability of paired serum and urine samples and written informed consent. Exclusion criteria included: the presence of other active malignancies, chronic inflammatory or autoimmune diseases, acute infections, or recent hospital admissions within four weeks prior to enrollment. Patients with severe renal impairment (estimated glomerular filtration rate < 30 mL/min/1.73 m^2^) or significant hepatic dysfunction (serum transaminases > 3× upper normal limit) were excluded to reduce potential confounding.

Baseline demographic data (age, sex), relevant clinical information, and routine hematological parameters were systematically recorded. Venous blood and urine samples were collected in the fasting state and prior to therapeutic intervention, ensuring that trace element determinations reflected the intrinsic metabolic state of the disease.

All serum and urine samples were collected before the initiation of any anticancer therapy. None of the enrolled patients had received neoadjuvant chemotherapy, radiotherapy, or surgical intervention at the time of sampling.

All procedures complied with the ethical principles of the Declaration of Helsinki (1964) and subsequent amendments. The study protocol was approved by the Independent Ethics Committee of the Azienda Ospedaliero-Universitaria di Cagliari (protocol code: PNCR_SS, version 5/05/2022; date of approval: 5 October 2022).

### 2.2. Analysis of Serum and Urinary Metals

Total concentrations of metals and metalloids in serum (aluminum, antimony, arsenic, barium, beryllium, cadmium, chromium, cobalt, copper, iron, lead, lithium, manganese, mercury, nickel, molybdenum, selenium, silver, thallium, tin, vanadium, and zinc) and urine (antimony, arsenic, barium, cadmium, cobalt, copper, iron, lead, manganese, mercury, molybdenum, selenium, vanadium, and zinc) were determined using inductively coupled plasma mass spectrometry (ICP-MS), following the US EPA 6020B method.

For each analysis, 0.5 mL of biological fluid was diluted in 5 mL of 2% ultrapure nitric acid (J.T. Baker, Phillipsburg, NJ, USA). Measurements were performed on an Agilent 8800 QQQ ICP-MS/MS system (Santa Clara, CA, USA), equipped with a collision/reaction cell and dual quadrupole mass analyzers, allowing accurate mass separation and effective interference removal compared with single-quadrupole configurations.

To correct for matrix effects and instrumental drift, internal standards were added to all samples. Calibration curves were verified at the beginning of each batch using independent Initial Calibration Verification (ICV) standards, and Continuous Calibration Verification (CCV) was performed at midrange concentrations. The limit of quantification (LOQ) was 0.001 ng/mL for all elements.

Quality assurance was ensured through the analysis of certified reference materials (ClinChek^®^ Urine Control and ClinChek^®^ Plasma Control for Trace Elements, RECIPE Chemicals, München, Germany). The laboratory participates in international proficiency testing schemes (OELM) and is accredited under UNI EN ISO 17025:2017.

### 2.3. Statistical Analysis

The statistical strategy combined univariate hypothesis testing with multivariate chemometric modeling to comprehensively evaluate differences in metal concentrations and their associations with disease status.

For univariate analysis, comparisons of individual serum and urinary metal concentrations between PDAC patients and control subjects were performed using the Wilcoxon rank-sum test, a non-parametric method suitable for independent groups with non-Gaussian distributions (Conover, Practical Nonparametric Statistics, ISBN: 978-0-471-16068-7).

Multivariate modeling was used to identify complex, multidimensional patterns discriminating PDAC from controls. All variables were first transformed using the Adaptive Box–Cox (ABC) procedure, which optimizes distributions towards normality and stabilizes variance [33,34]. Data were then autoscaled to normalize magnitudes and ensure equal weighting. Principal Component Analysis (PCA) was applied for dimensionality reduction, and Linear Discriminant Analysis (LDA) was subsequently performed for supervised classification.

The optimal number of principal components (PCs) was determined by 5-fold cross-validation to balance accuracy and model complexity. Robustness was further evaluated using Monte Carlo cross-validation (1000 iterations), with 30% of samples randomly assigned to the test set at each iteration. Classification accuracy across iterations provided stringent validation of model reliability.

All analyses were conducted in MATLAB 2024b (MathWorks, Natick, MA, USA) using the Milano Chemometrics and QSAR Toolbox (v.7.0). Graphical outputs were generated in R 4.3.1 (R Foundation for Statistical Computing, Vienna, Austria) [35].

## 3. Results

### 3.1. Univariate Analysis

The study included 71 patients with pancreatic ductal adenocarcinoma (PDAC) and 70 healthy controls. The median age of PDAC patients was 75 years (range 48–93), with a mean of 71.4 ± 9.5 years. The control group had a median age of 61 years (range 20–87) and a mean of 57.0 ± 18.9 years. Sex distribution in the PDAC cohort was balanced, with 36 females (50.7%) and 35 males (49.3%), while the control cohort showed the same proportions.

**Figure 1:**
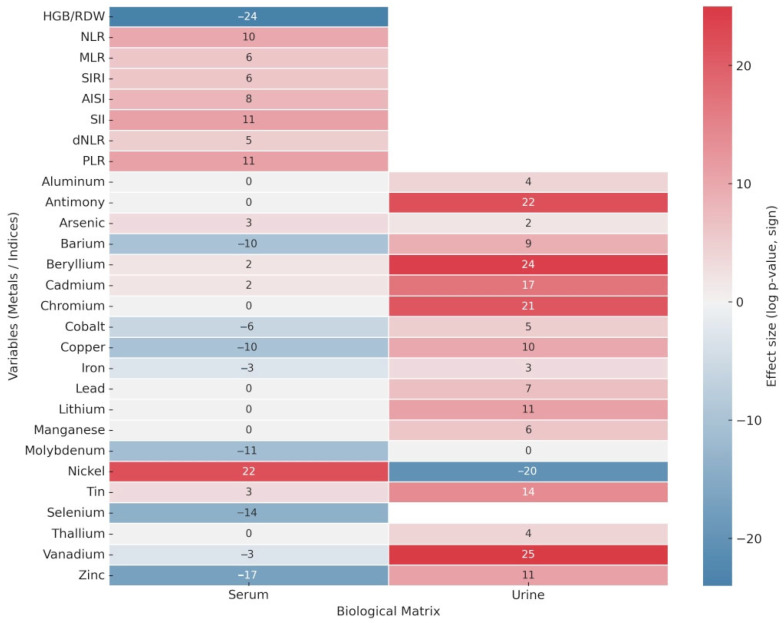
Heatmap showing differential serum and urinary metal concentrations and hematological inflammatory indices between PDAC patients and controls (Wilcoxon test).

Since most variables exhibited markedly non-Gaussian distributions, comparisons between groups were performed using the non-parametric two-sided Wilcoxon rank-sum test [36]. The results, summarized in Table 1 and visualized in Figure 1, revealed a greater number of statistically significant alterations in urine compared with serum. Notice that only markers displaying a significant difference in either serum or urine are reported in Table 1. Patients with PDAC showed markedly elevated urinary concentrations of antimony, chromium, cadmium, and vanadium (all *p* < 10^−5^), findings that suggest disease-related disturbances in detoxification and excretion pathways. Additional urinary elements, including copper, iron, nickel, and beryllium, also differed significantly between cases and controls.

In contrast, serum profiles indicated that selenium, zinc, barium, vanadium, and cobalt were significantly higher in controls (*p* < 10^−5^). These results are consistent with a potential protective or homeostatic role of these elements, whereas their reduction in PDAC patients may reflect impaired absorption, redistribution to tumor tissues, or metabolic reprogramming associated with the disease. Notably, some elements demonstrated opposing trends across matrices. Zinc, for example, was elevated in serum from controls but increased in urine from PDAC patients, a finding that may indicate enhanced turnover or urinary loss. Nickel showed the opposite behavior, being elevated in the serum of PDAC patients but higher in urine from controls, pointing to differences in systemic regulation of this element.

Analysis of hematological inflammatory indices further confirmed the link between PDAC and systemic inflammation. Markers such as NLR, MLR, SIRI, AISI, SII, dNLR, and PLR were significantly elevated in PDAC patients, whereas HGB/RDW was higher in controls.

The regulation levels of metals and inflammatory indices highlighted in Table 1 are in line with the median of each variable associated with the two clinical classes, whose values are reported in the Supplementary Materials (Table S1). Taken together, these findings delineate a dual biochemical pattern in PDAC characterized by the depletion of protective serum trace elements, including selenium, zinc, cobalt, and barium, and by the increased urinary excretion of toxic or pro-oxidant metals, such as antimony, chromium, cadmium, and vanadium. This composite signature reflects systemic oxidative stress and disrupted metal homeostasis in PDAC and supports the role of these variables as candidate biomarkers for subsequent multivariate analyses.

### 3.2. Multivariate Analysis (PCA–LDA)

**Figure 2:**
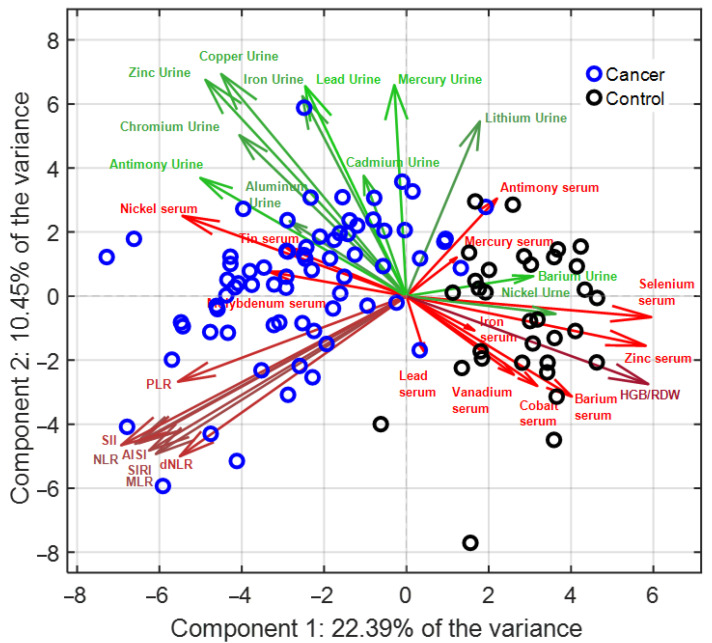
PCA Biplot: Scores related to cancer and control cohort are reported in blue and black, respectively. Loadings related to metal content within urines are reported in green, whereas those pertaining to serum are in red. Finally, inflammatory markers are highlighted in brown. Uninformative loading values were removed to avoid the plot weighting.

To explore multidimensional associations and identify discriminative biochemical signatures, all variables were pre-processed using the ABC transformation to approximate normality, followed by autoscaling to equalize variance contribution across features. Lilliefors’ normality test was applied to find the optimal value of ABC transformation parameter. The outcomes of such transformation are reported in the Supplementary Table S2. PCA was first applied to reduce dimensionality and provide an overview of the underlying data structure, and LDA was then employed for supervised classification. The optimized PCA–LDA model, based on four PCs, accounted for 46% of the total variance and achieved 100% classification accuracy in 5-fold cross-validation. Robustness was further confirmed through Monte Carlo cross-validation, in which 1000 iterations with 30% randomly selected test sets yielded an average classification accuracy of 99%. Detailed results of transformation parameters and normality testing are provided in Supplementary Table S3, while cross-validation error profiles and Monte Carlo validation plots are reported in Supplementary Figure S1. The PCA biplot (Figure 2) demonstrated a clear separation between PDAC patients and controls along the first PC, while the second component captured secondary variance. Within the multivariate analysis, a small subset of variables (e.g., serum beryllium, chromium, cadmium or thallium) was discarded due to the high sparsity of the relevant vectors which may distort the loading values if considered for the PC computation. A greater dispersion of PDAC cases was evident, reflecting biological heterogeneity within the group, which may be related to disease stage or inter-individual metabolic variation. Examination of loading vectors highlighted that serum selenium, zinc, barium, vanadium, and cobalt were strongly associated with controls, whereas serum nickel clustered with PDAC. By contrast, urinary elements such as antimony, chromium, zinc, copper, and iron were aligned with PDAC, while urinary nickel correlated with controls. Hematological indices tended to cluster together and were largely orthogonal to the axis separating PDAC from controls, with the exception of HGB/RDW, which was more closely associated with the control group. This multivariate approach therefore confirmed the discriminatory power of combined metallomic and inflammatory profiles, demonstrating that serum selenium and zinc, along with urinary antimony and chromium, are central contributors to the separation between PDAC and controls, and highlighting their potential as candidate biomarkers for diagnostic applications.

### 3.3. Correlations with Inflammatory Indices

**Figure 3:**
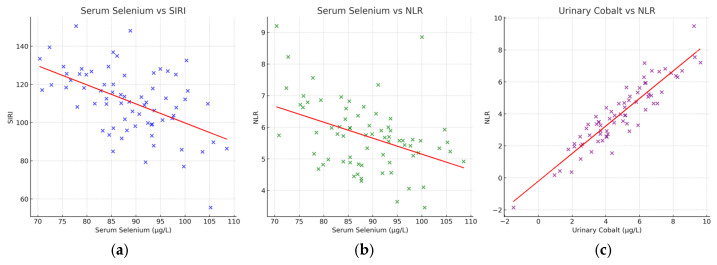
Scatterplots of correlations between trace elements and inflammatory indices in PDAC. (**a**) Serum selenium vs. SIRI, (**b**) serum selenium vs. NLR, and (**c**) urinary cobalt vs. NLR. Red dashed lines indicate regression fits.

Correlation analyses were performed to investigate the relationship between trace element concentrations and systemic inflammatory markers. As shown in Figure 3, serum selenium exhibited significant inverse correlations with both the systemic inflammation response index (SIRI) and the neutrophil-to-lymphocyte ratio (NLR) (*p* < 0.05). These findings indicate that selenium depletion in PDAC patients is closely associated with heightened systemic inflammation. In contrast, urinary cobalt demonstrated a positive correlation with NLR (*p* < 0.05), suggesting that increased cobalt excretion may reflect or contribute to pro-inflammatory states. Taken together, these results provide further evidence of the biochemical link between altered trace element regulation and systemic inflammation in PDAC and reinforce their potential utility as integrated diagnostic and prognostic biomarkers.

## 4. Discussion

This study provides novel insights into the systemic metallomic alterations associated with pancreatic ductal adenocarcinoma (PDAC), demonstrating profound imbalances in the regulation of essential and toxic elements. The results revealed a dual biochemical pattern, with marked depletion of protective serum trace elements, most notably selenium and zinc—and enhanced urinary excretion of toxic or pro-oxidant metals such as cadmium, chromium, vanadium, and antimony. These findings are consistent with previous observations of trace element dysregulation in pancreatic cancer [1,2,3,4] and reinforce the concept that PDAC progression is closely linked to systemic metal dyshomeostasis. The inverse patterns detected in serum and urine suggest impaired retention of micronutrients on one hand, and mobilization or detoxification of carcinogenic metals on the other, likely reflecting tumor-driven metabolic reprogramming and systemic stress responses. Among the trace elements investigated, selenium emerged as a central biomarker. Its pronounced depletion in serum, together with inverse correlations with systemic inflammatory indices such as neutrophil-to-lymphocyte ratio (NLR) and systemic inflammation response index (SIRI), highlights its pivotal role in redox balance. Selenium is an essential cofactor for glutathione peroxidases and thioredoxin reductases, enzymes that neutralize reactive oxygen species (ROS) and prevent lipid peroxidation and DNA damage [5,6]. A deficiency in selenium may therefore compromise antioxidant defenses, promote genomic instability, and facilitate tumor progression. These findings align with clinical evidence showing that low selenium status is associated with poor outcomes in gastrointestinal and pancreatic cancers, whereas higher selenium levels correlate with improved survival [7,8]. Beyond its antioxidant functions, selenium deficiency may further amplify systemic inflammation, creating a feed-forward loop between oxidative stress and pro-inflammatory signaling.

In contrast, toxic metals were consistently elevated in urine from PDAC patients, pointing to altered systemic handling or increased body burden. Cadmium, a Group 1 carcinogen, exemplifies this trend. Mechanistically, cadmium contributes to carcinogenesis by generating ROS, depleting glutathione, and displacing zinc in zinc-finger motifs of DNA repair proteins, thereby impairing repair mechanisms such as nucleotide excision repair [9,10]. It also induces aggregation of repair proteins like BLM helicase, resulting in persistent DNA breaks and genomic instability [11], and has been linked to epigenetic dysregulation, including promoter hypermethylation of tumor suppressor genes and altered microRNA expression [12]. The markedly elevated urinary cadmium levels observed in PDAC patients may represent an attempt at elimination, albeit at the cost of sustained genotoxic stress. Chromium and vanadium showed similar patterns, supporting their role in oxidative DNA damage and epigenetic remodeling [13,14]. Nickel presented divergent behavior, being elevated in serum of PDAC patients but higher in urine from controls. Although chronic nickel exposure is recognized as carcinogenic through inhibition of histone demethylases and induction of promoter hypermethylation [15], some epidemiological studies have paradoxically linked higher nickel levels to reduced PDAC risk [16]. These discrepancies underscore the complexity of nickel biology and warrant further mechanistic investigation in pancreatic cancer models. Importantly, multivariate chemometric modeling demonstrated that composite metallomic signatures offer strong discriminatory power between PDAC patients and controls. PCA–LDA achieved ~99% accuracy, with selenium, zinc, and cobalt clustering with controls, while cadmium, chromium, and vanadium aligned with PDAC. These findings are consistent with previous work highlighting the sensitivity of urinary metallomic panels for PDAC detection [17] and emphasize the added value of multidimensional data integration in uncovering latent biochemical patterns beyond the scope of individual biomarkers. Hematological inflammatory indices clustered independently of the metallomic axis, although significant correlations—such as the inverse association between selenium and NLR, and the positive association between urinary cobalt and NLR—point to meaningful crosstalk between trace element regulation and systemic inflammation. Elevated urinary cobalt in inflamed patients may reflect hypoxia–inflammation interactions, given the known role of cobalt ions in stabilizing HIF-1α and amplifying inflammatory signaling [18].

The prognostic relevance of systemic inflammation in PDAC has been increasingly recognized in recent years. Zhang et al. first demonstrated that the systemic immune-inflammation index (SII), derived from platelet, neutrophil, and lymphocyte counts, serves as an independent predictor of overall and progression-free survival in patients with advanced pancreatic cancer [37]. In a comprehensive single-center analysis of 1294 PDAC patients, Neumann et al. confirmed that several hematological inflammation-based scores—namely, the neutrophil-to-lymphocyte ratio (NLR), lymphocyte-to-monocyte ratio (LMR), C-reactive protein-to-albumin ratio (CAR), and the novel inflammatory benchmark index (IBI)—were all independent prognostic markers for overall survival, regardless of tumor stage or treatment intent [38]. These findings have been corroborated by a large meta-analysis of digestive system carcinomas by Niu et al., which established the systemic inflammation response index (SIRI) as a robust and reproducible prognostic biomarker (HR ≈ 2.0 for overall survival). Collectively, these studies highlight the strong prognostic value of inflammatory indices as accessible surrogates of tumor–host interactions and immune dysregulation in PDAC [39]. In the context of our results, the inverse correlations between selenium and both NLR and SIRI suggest a biochemical link between redox imbalance and systemic inflammatory burden. Given that low selenium levels may amplify oxidative stress and promote chronic inflammation, our data support the hypothesis that trace element imbalance and inflammation act as convergent drivers of disease progression. Integrating metallomic signatures with established inflammatory indices may therefore improve risk stratification, prognostic assessment, and the design of personalized therapeutic strategies targeting both oxidative and inflammatory pathways in PDAC.

From a clinical biochemical perspective, these results highlight the potential of combined metal–inflammation signatures for patient stratification. Unsupervised clustering identified PDAC subgroups with distinct biochemical profiles, ranging from relatively preserved selenium status and lower inflammation to profound selenium depletion accompanied by increased urinary chromium and zinc excretion and the highest inflammatory burden. This stratification mirrors the clinical heterogeneity of PDAC and suggests that biochemical markers may help identify patients at risk of aggressive disease trajectories, systemic inflammation, and cachexia. Such integrative approaches align with the principles of precision oncology, opening the possibility of targeted interventions including micronutrient supplementation or anti-inflammatory strategies in selected subgroups. The translational implications of this work are significant. Metallomic profiling may complement existing diagnostic tools such as CA19-9, which is limited by poor sensitivity and specificity. Trace element signatures in serum and urine, particularly selenium depletion and urinary cobalt excretion, could serve as non-invasive adjunct markers for early detection and disease monitoring. Moreover, parameters such as the copper-to-zinc ratio, although not directly examined here, have been reported as prognostic indicators in other malignancies and merit investigation in PDAC. Longitudinal monitoring of metallomic changes could also provide valuable insights into therapeutic response and disease progression. This study has limitations. The cross-sectional design precludes causal inference, and the absence of dietary or environmental exposure data limits the interpretation of metal origins. Furthermore, although the sample size was adequate for exploratory chemometric analysis, validation in larger, multi-center cohorts is essential, as is the evaluation of specificity against differential diagnoses such as chronic pancreatitis. Mechanistic studies using cell-based and animal models will be necessary to clarify the biological consequences of selenium depletion and toxic metal accumulation. Finally, multi-omics approaches integrating metallomics with genomics, transcriptomics, proteomics, and metabolomics could provide a more comprehensive understanding of how trace element imbalance interfaces with PDAC metabolic reprogramming, immune evasion, and tumor microenvironment dynamics.

## 5. Conclusions

Pancreatic ductal adenocarcinoma is associated with systemic trace element imbalances, characterized by serum depletion of protective micronutrients such as selenium and zinc and increased urinary excretion of toxic metals including cadmium, chromium, vanadium, and antimony. These alterations correlate with systemic inflammation, underscoring the interplay between metal dyshomeostasis and tumor-driven inflammatory responses. ICP-MS–based metallomic profiling, combined with chemometric modeling, provided robust diagnostic discrimination between PDAC patients and controls. Selenium depletion and urinary cobalt emerged as candidate biomarkers with potential diagnostic and prognostic value. Metallomic–inflammatory signatures may therefore complement existing tools such as CA19-9 and support early detection, patient stratification, and personalized management in PDAC. Validation in larger, longitudinal studies will be critical to translate these insights into clinical practice and establish metallomic profiling as a complementary tool in oncology diagnostics.

## Figures and Tables

**Table 1:** Wilcoxon rank-sum tests comparing serum and urinary metal concentrations and hematological indices between PDAC patients (*n* = 71) and controls (*n* = 69). Direction of effect is shown with colors (red PDAC/green Controls). NA, not available.

Haemotological Data	*p*-Values	
HGB/RDW	6.28 × 10^−24^	
NLR	1.13 × 10^−10^	
MLR	2.65 × 10^−6^	
SIRI	1.59 × 10^−6^	
AISI	8.51 × 10^−8^	
SII	1.73 × 10^−11^	
dNLR	1.02 × 10^−5^	
PLR	9.35 × 10^−11^	
**Metals**	**Serum *p*-values**	**Urine *p*-values**
Aluminum		1.96 × 10^−4^
Antimony		2.54 × 10^−22^
Arsenic	7.46 × 10^−3^	4.45 × 10^−2^
Barium	8.08 × 10^−10^	2.07 × 10^−9^
Beryllium	3.53 × 10^−2^	1.04 × 10^−24^
Cadmium	3.87 × 10^−2^	1.99 × 10^−17^
Chromium		9.39 × 10^−21^
Cobalt	5.37 × 10^−6^	8.11 × 10^−5^
Copper		1.53 × 10^−10^
Iron	3.82 × 10^−3^	1.22 × 10^−3^
Lead		4.92 × 10^−7^
Lithium		1.29 × 10^−11^
Manganese		9.46 × 10^−6^
Molybdenum	5.02 × 10^−11^	
Nickel	2.31 × 10^−22^	5.17 × 10^−20^
Tin	8.83 × 10^−3^	2.25 × 10^−14^
Selenium	3.73 × 10^−14^	NA
Thallium		1.03 × 10^−4^
Vanadium	4.25 × 10^−3^	6.03 × 10^−25^
Zinc	2.69 × 10^−17^	1.24 × 10^−11^

	No statistically significant difference (*p* ≥ 0.05)	
	Significantly higher in the cancer group (*p* < 10^−5^)	
	Significantly higher in the cancer group (*p* < 0.05)	
	Significantly higher in the control group (*p* < 0.05)	
	Significantly higher in the control group (*p* < 10^−5^)	

## Data Availability

The data presented in this study are available on reasonable request from the corresponding author.

## Supplementary Materials

The following supporting information can be downloaded at: https://www.mdpi.com/article/10.3390/diagnostics15212818/s1, Material S1: Measures of central tendencies for the investigated variables; Table S1: Class median values for each variable investigated in the study; Material S2: ABC transformation of raw data; Table S2: Lilliefors test *p*-values assessing normality within the cancer (p_1_) and control (p_2_) groups, together with the corresponding ABC transformation parameter (λ) applied to each variable; Material S3: Hyperparameters optimization; Figure S1: Error rate in 5-fold cross-validation (1-accuracy) for the PCA-LDA model as a function of the number of PCs; Material S4: Analysis of Covariance including age as continuous covariate; Table S3: Results of ANCOVA comparing serum and urinary metal concentrations and hematological indices between PDAC patients and controls, with age included as a covariate.

## Abbreviations

The following abbreviations are used in this manuscript:

Abbreviation	Definition
ABC	Adaptive Box–Cox
AISI	Aggregate Index of Systemic Inflammation
BLM	Bloom syndrome protein (DNA helicase)
CA19-9	Carbohydrate Antigen 19-9
Cd	Cadmium
Cr	Chromium
Cu	Copper
Fe	Iron
HGB/RDW	Hemoglobin/Red Cell Distribution Width ratio
ICP-MS	Inductively Coupled Plasma Mass Spectrometry
LDA	Linear Discriminant Analysis
MLR	Monocyte-to-Lymphocyte Ratio
Mn	Manganese
Na	Not available
Ni	Nickel
NLR	Neutrophil-to-Lymphocyte Ratio
PCA	Principal Component Analysis
PDAC	Pancreatic Ductal Adenocarcinoma
PLR	Platelet-to-Lymphocyte Ratio
QC	Quality Control
ROS	Reactive Oxygen Species
Sb	Antimony
Se	Selenium
SII	Systemic Immune-Inflammation Index
SIRI	Systemic Inflammation Response Index
Sn	Tin
V	Vanadium
Zn	Zinc
