# Supplementary material for: Diagnostic Stratification of Pancreatic Ductal Adenocarcinoma via Metallomics and Blood-Based Biomarkers
Source: Diagnostics (Basel). 2025 Nov 6;15(21):2818. doi: 10.3390/diagnostics15212818 (PMC12610246; doi:10.3390/diagnostics15212818)
Supplement: Supplementary file 1 [file diagnostics-15-02818-s001.zip › diagnostics-3915317-supplementary.pdf]

Supplementary materials

Material S1. Measures of central tendencies for the investigated variables

In order to highlight the key quantitative differences between the control and cancer groups, regulation of the variables was taken into consideration. Table S1 shows the median values for the control and cancer groups, respectively, from which it can be seen that regulation of the variables is consistent with the outcomes reported in the main body of the text.

Table S1 Class median values for each variable investigated in the study.

Variable	Median Control	Median Cancer	Variable	Median Control	Median Cancer	Variable	Median Control	Median Cancer
Lithium Serum	2.4	3.5	Chromium Serum	0	0	Vanadium Urine	0	0.26
Manganese Serum	1.7	2	Lead Serum	0.1	0.2	Antimony Urine[b]	0	0.45
Cobalt Serum[a]	0.3	0.2	Cadmium Serum[b]	0	0	Barium Urine[a]	1.9	1.09
Vanadium Serum[a]	0.1	0.1	Lithium Urine[a]	50	20.14	Thallium Urine[a]	0.2	0.13
Iron Serum[a]	998	786	Aluminum Urine[b]	8.4	11.99	Lead Urine[b]	0.5	1.09
Nickel Serum[b]	0	1.6	Manganese Urine[a]	4	2.75	Beryllium Urine[b]	0	0.05
Copper Serum	1063	1145	Cobalt Urine[a]	0.5	0.36	Tin Urine[b]	0.2	1.09
Zinc Serum[a]	1259	786	Chromium Urine[b]	0.2	1.15	HGB/RDW[a]	3.03	0.81
Selenium Serum[a]	111	76	Iron Urine[b]	17.8	26.07	NLR[b]	1.5	2.6
Arsenic Serum[b]	0.6	0.9	Nickel Urine[a]	3.7	1.99	MLR[b]	0.2	0.32
Molybdenum Serum[b]	0.6	1.2	Copper Urine[b]	9.4	27.07	SIRI[b]	0.59	1.23
Tin Serum	0.2	0.2	Zinc Urine[b]	327	1266.66	AISI[b]	142.66	350.96
Antimony Serum	1.3	1.4	Selenium Urine	*	43.41	PCT	0.26	0.24
Barium Serum[a]	2.3	1.3	Arsenic Urine[b]	12.8	39.87	SII[b]	310.76	750.88
Beryllium Serum[a]	0	0	Molybdenum Urine	43.7	42.17	dNLR[b]	1.25	1.71
Thallium serum	0	0	Cadmium Urine[b]	0.3	1.75	PLR[b]	92.7	167

*Not enough Data; [a] Upregulated in control group; [b] Upregulated in cancer group

Material S2. ABC transformation of raw data

Table S2 reports the p-values obtained from the Lilliefors test for normality performed separately within the cancer (p_1) and control (p_2) groups for each variable. In addition, the table includes the corresponding λ parameter used in the ABC transformation, applied to stabilize variance and improve normality where needed.

Table S2. Lilliefors test p-values assessing normality within the cancer (p_1) and control (p_2) groups, together with the corresponding ABC transformation parameter (λ) applied to each variable.

Variable	p1	p2	Λ
Lithium Serum	0.001	0.009	0.66
Manganese Serum	0.456	0.061	0.68

Cobalt Serum	0.001	0.001	0.01
Vanadium Serum	0.001	0.001	0.01
Iron Serum	0.029	0.142	0.04
Nickel Serum	0.007	0.001	0.45
Copper Serum	0.353	0.500	0.45
Zinc Serum	0.368	0.500	0.17
Selenium Serum	0.500	0.066	0.59
Arsenic Serum	0.016	0.001	0.15
Molybdenum Serum	0.033	0.006	0.51
Tin Serum	0.001	0.001	0.01
Antimony Serum	0.015	0.026	0.32
Barium Serum	0.395	0.066	-0.36
Mercury Serum	0.309	0.500	0.2
Lead Serum	0.001	0.001	0.01
Lithium Urine	0.001	0.001	-3
Aluminum Urine	0.001	0.236	-0.04
Manganese Urine	0.001	0.500	-0.15
Cobalt Urine	0.001	0.019	-0.24

Chromium Urine	0.001	0.001	0.01
Iron Urine	0.001	0.402	0.29
Nickel Urine	0.001	0.500	-0.73
Copper Urine	0.001	0.500	0.39
Zinc Urine	0.001	0.500	0.3
Selenium Urine	0.001	0.179	0.5
Arsenic Urine	0.001	0.001	0.01
Molybdenum Urine	0.001	0.181	0.51
Cadmium Urine	0.001	0.001	0.01
Tin Urine	0.001	0.001	0.01
Antimony Urine	0.001	0.001	0.01
Barium Urine	0.001	0.359	0.15
Mercury Urine	0.001	0.029	0.17
Thallium Urine	0.001	0.001	-3
Lead Urine	0.001	0.289	0.52
HGB/RDW	0.009	0.067	3
NLR	0.500	0.006	-0.66
MLR	0.398	0.171	-0.06
SIRI	0.500	0.500	-0.31
AISI	0.500	0.500	-0.16
PCT	0.499	0.243	0.36

SII	0.500	0.356	-0.04
dNLR	0.500	0.130	-0.15
PLR	0.500	0.500	-0.56

Material S3. Hyperparameters optimization

Figure S1 shows the cross-validation error rate profile over model complexity. Despite one PC being sufficient to achieve satisfactorily classification performances, the achievement of 100% accuracy was obtained from a 4 PC model. The use of more complex models did not significantly change the model performances (Figure S1).

For the sake of a robust assessment of the model's discriminatory capacity, the PCA-LDA model with 4 PCs was further validated through Monte Carlo cross-validation with 1000 iterations. In this case, 99% accuracy was obtained, indicating high classificatory performance of PCA-LDA.

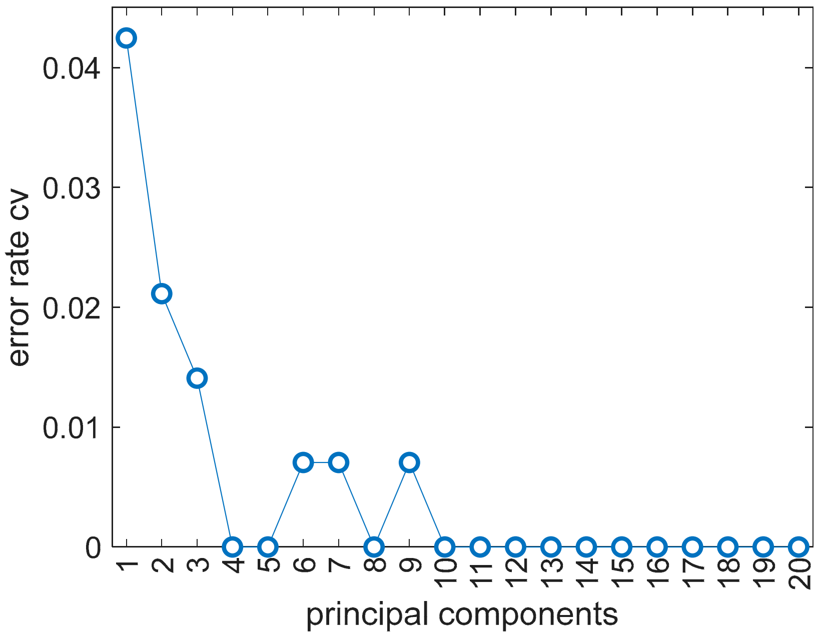

Figure S1. Error rate in 5-fold cross-validation (1-accuracy) for the PCA-LDA model as a function of the number of PCs.

Material S4. Analysis of Covariance including age as continuous covariate

To account for the potential confounding effect of age, an Analysis of Covariance (ANCOVA) was performed, including disease status as a categorical factor and age as a continuous covariate. Because ANCOVA assumes normally distributed data, the ABC-transformed variables were used in the analysis. The results of this age-adjusted model are

presented in Table S3 and indicate that the main findings remain consistent after adjustment for age.

Table S3: Results of ANCOVA comparing serum and urinary metal concentrations and hematological indices between PDAC patients and controls, with age included as a covariate.

Species	p-value Group	p-value Age
Antimony Serum	9.71E-01	4.89E-01
Arsenic Serum	2.92E-01	5.74E-01
Barium Serum **	7.17E-07	7.85E-01
Cobalt Serum *	2.03E-04	5.66E-01
Copper Serum⁺	4.97E-01	1.03E-02
Iron Serum	6.80E-01	8.28E-02
Lead Serum	8.47E-01	8.31E-02
Lithium Serum	5.28E-01	9.98E-01
Manganese Serum	2.21E-01	9.41E-01
Molybdenum Serum **	3.92E-09	8.34E-01
Nickel Serum **	7.23E-16	4.76E-01
Selenium Serum **	2.93E-13	4.01E-01
Tin Serum	3.18E-01	8.54E-01
Vanadium Serum	6.82E-01	7.11E-01
Zinc Serum **	1.97E-15	7.89E-01
HGB/RDW**	1.94E-78	5.77E-01
NLR*	6.07E-04	5.89E-01
MLR*	2.04E-04	6.18E-01
SIRI*	2.77E-02	7.30E-01
AISI*	1.14E-02	5.34E-01
PCT	7.21E-01	1.35E-01
SII*	1.52E-04	9.72E-01
dNLR*	3.58E-03	1.10E-01
PLR**	1.31E-08	9.74E-01

Species	p-value Group	p-value Age
Aluminum Urine	1.94E-01	3.45E-01
Antimony Urine*	2.13E-03	9.18E-01
Arsenic Urine	9.97E-01	7.72E-01
Barium Urine**	2.01E-07	1.70E-01
Cadmium Urine**	2.65E-08	5.69E-01
Chromium Urine**	2.04E-11	7.54E-01
Cobalt Urine*	1.17E-02	3.71E-01
Copper Urine**	1.04E-05	6.41E-01
Iron Urine**	1.33E-03	3.02E-01
Lead Urine*	3.66E-05	5.57E-01
Lithium Urine**	3.74E-10	1.90E-01
Manganese Urine**,⁺	2.89E-08	1.08E-02
Molybdenum Urine	7.59E-01	7.70E-01
Nickel Urine**	8.53E-22	7.77E-01
Selenium Urine	3.33E-01	6.13E-01
Tallium Urine**	4.66E-06	1.19E-01
Tin Urine*	2.90E-05	3.16E-01
Vanadium Urine*	4.29E-02	9.50E-01
Zinc Urine**	1.05E-08	7.18E-01

Significant difference between the groups: * p-value < 0.05, ** p-value <10e-5

Significant dependence on the age: ⁺ p-value < 0.05

Only serum copper and urinary manganese show a slightly significant time dependence. Regarding the group comparisons, the main findings obtained with the Wilcoxon test were confirmed, except for a few variables highlighted in red in the Table. These discrepancies

likely stem from the limited ability of the ABC transformation to normalize some variables (see Table S2). Although the transformation was optimized to maximize the objective function [1,2], several variables still showed low optimal p-values, indicating residual deviations from normality. Such deviations may explain their inconsistent behavior in the ANCOVA model.

[1] Sibono, L., M. Grosso, E. Tejedor-Calvo, M. Casula, P. Marco-Montori, S. Garcia-Barreda, C. Manis and P. Caboni. "A critical analysis of adaptive box-cox transformation for skewed distributed data management: Metabolomics of spanish and argentinian truffles as a case study." Analytica Chimica Acta 1345 (2025): 343704.

[2] Yu, H., P. Sang and T. Huan. "Adaptive box–cox transformation: A highly flexible feature-specific data transformation to improve metabolomic data normality for better statistical analysis." Analytical Chemistry 94 (2022): 8267-76.
